# First-line albumin-bound paclitaxel/carboplatin plus apatinib in advanced pulmonary sarcomatoid carcinoma: A case series and review of the literature

**DOI:** 10.1097/MD.0000000000020667

**Published:** 2020-06-05

**Authors:** Feng-Wei Kong, Wei-Min Wang, Lei Liu, Wen-Bin Wu, Xiang Wang, Miao Zhang

**Affiliations:** a Department of General Surgery, Xuzhou Infectious Disease Hospital; b Department of Gastroenterology, Yichang Central People's Hospital, Institute of Digestive Disease, China Three Gorges University, Yichang; c Department of Surgery, Xuzhou Central Hospital, Xuzhou, China.

**Keywords:** anti-angiogenesis, apatinib, immunotherapy, paclitaxel, pulmonary sarcomatoid carcinoma, targeted therapy

## Abstract

**Rationale:**

Pulmonary sarcomatoid carcinoma (PSC) is an uncommon type of non-small cell lung cancer, exhibiting aggressive behavior and resistance to the conventional chemoradiotherapy. To date, the optimal treatment for PSC has not been elucidated.

**Patient concerns:**

Three male patients including a 69-year-old smoker (Case 1), a 45-year-old non-smoker (Case 2), and a 69-year-old smoker (Case 3) were admitted because of cough, back pain, and loss of body weight respectively.

**Diagnoses:**

Radiographical examinations in these patients showed bulky intrathoracic lesions, which were pathologically diagnosed as PSC staging III–IV by computed tomography–guided percutaneous biopsy and endoscopy.

**Interventions:**

Immunotherapy was not covered by their health insurance and they refused immune checkpoint inhibitors for financial reasons. In addition, a radical resection was not appropriate due to the advanced staging of these lesions. Therefore, first-line albumin-bound paclitaxel (nab-paclitaxel, 260 mg/m^2^ of the body surface area) and carboplatin (area under curve 5) combined with oral apatinib (425 mg, daily) were administered empirically.

**Outcomes:**

Two patients achieved a partial response and the other case showed stable disease lasting for more than 6 months. However, 1 of them indicated progression on the 7-month follow up.

**Lessons:**

Nab-paclitaxel/carboplatin plus apatinib showed limited short-term efficacy in advanced, unresectable PSC. The rapid resistance of PSC to the current therapeutic regimen necessitates further researches, as more effective agents are urgently needed.

## 1 Introduction

Pulmonary sarcomatoid carcinoma (PSC) is a rare subtype of non-small-cell lung cancer (NSCLC), which has aggressive behavior with dismal prognosis. No consensus in the treatment protocol for this refractory disease has been established, and new therapeutic strategies are urgently needed because of the limited efficacy of surgery and conventional chemoradiotherapy.

Apatinib is reported to be effective in advanced sarcoma,^[1]^ and is also effective for advanced NSCLC that failed prior chemotherapy.^[2]^ The efficacy of albumin-bound paclitaxel (nab-paclitaxel) and carboplatin combined with apatinib in PSC has not been reported before. Herein, we presented 3 PSC patients who received this treatment regimen and obtained a median progression-free survival of more than 6 months. Furthermore, the updated literatures regarding the management of PSC were reviewed briefly.

## 2 Case presentation

### 2.1 Case 1

**Figure 1:**
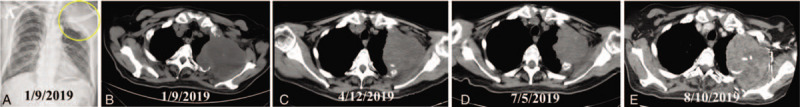
Radiological images of the pulmonary sarcomatoid carcinoma (indicated by arrow) before and after treatment in case 1. A. The X-ray showed a bulky mass located in left upper thorax on admission. B. Further computed tomography revealed the pulmonary tumor invading adjacent ribs. C. The tumor showed PR after 3 months of treatment. D. The tumor was shorter partial remission after 6 months of treatment. E. The lesion was significantly enlarged on the 7-month follow up. CT = computed tomography, PR = partial remission.

A 69-year-old male was hospitalized in January, 2019 because of cough and gradually aggravated left-sided chest pain in the previous 2 months. He had a smoking history of nearly 80 pack-years. The serum tumor biomarkers of neuron-specific enolase, cytokeratin-19 fragment, carcinoembryonic antigen, and pro-gastrin-releasing peptide were all in normal range. Then the chest X-ray and computed tomography (CT) showed locally advanced pulmonary tumor approximately 111 mm × 114 mm, invading parietal pleura and adjacent ribs (Fig. 1). V-Ki-ras2 Kirsten rat sarcoma viral oncogene homolog (KRAS), epidermal growth factor receptor , and echinoderm microtubule-associated protein-like 4-anaplastic lymphoma kinase (EML4-ALK) were not identified in the specimen by immunohistochemistry (IHC) except positive expression of programmed death ligand-1 (PD-L1). Positron emission tomography was not carried out as it was not covered by his health insurance. Bone emission CT, emission computed tomography and cranial magnetic resonance imaging scan excluded other distant metastases. PSC was confirmed by CT-guided fine-needle biopsy, staging as T4NxM1 (IV) according to the 8^th^ edition of the AJCC/UICC TNM staging system for lung cancer.^[3]^ Then he received first-line chemotherapy using carboplatin (Qilu Pharmaceutical Co., Ltd., China; AUC 5, day 1) and nab-paclitaxel (Abraxane, American Pharmaceutical Partners. Inc, Melrose Park, Illinois, 260 mg/m^2^ of body surface area, day 1 and 8) every 3 weeks for 4 cycles, in combination with oral apatinib (Jiangsu Hengrui Medicine Co., Ltd., China) at a dosage of 425 mg daily, with tolerable adverse events (AEs). In addition, zoledronic acid for injection (Jiangsu Hengrui Medicine Co., Ltd., China; 4 mg, once every month) was administered. Apatinib was continued as maintenance therapy thereafter until uncontrolled AEs or progressive disease. The efficacy was evaluated according to Response Evaluation Criteria in Solid Tumors 1.1. Partial remission of the tumor was indicated in the first 6 months since the treatment. Grade 2 thrombocytopenia, and Grade 3 hand-foot syndrome, according to National Cancer Institute Common Terminology Criteria for Adverse Events version 4.0, were observed and controlled effectively. However, the lesion was significantly enlarged on the 7-month follow up. Then apatinib was discontinued. However, the patient was not suitable to be involved in an immunotherapy trial because of his compromised performance status. Therefore, best supportive care was started as palliative treatment.

### 2.2 Case 2

**Figure 2:**
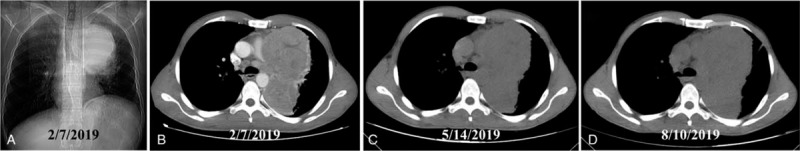
The bulky pulmonary lesion (indicated by arrow) before and after treatment in case 2. A. The X-ray showed a giant mass located in left upper thorax. B. Computed tomography revealed the pulmonary tumor invading adjacent pulmonary veins. C. The tumor showed stable disease after 3 months of treatment. D. The lesion remained stable 6 months later. SD = stable disease.

A 45-year-old male non-smoker was admitted on February, 2019 with severe pain of the left back for 1 month. Serum tumor markers including carcinoembryonic antigen , and neuron-specific enolase were within the normal range. CT revealed a giant soft mass (139 × 78 mm) located in the left upper lung, invading the adjacent pulmonary veins and mediastinum (Fig. 2). Then locally advanced PSC staging T4NxM0 (III) was diagnosed by fine-needle biopsy under bronchoscopy. The specimen was negative of epidermal growth factor receptor, ALK, KRAS, ROS proto-oncogene 1 (ROS1), human epidermal growth factor receptor-2 (HER2), and rearranged during transfection proto-oncogene (RET), except PD-L1 (>95%) by IHC. Nab-paclitaxel/carboplatin and apatinib (425 mg, daily) was administered for 4 cycles. On the 6-month follow up, the tumor remained stable. The major AEs were Grade 2 thrombocytopenia, and hypertension, without hemoptysis. His progression-free survival and overall survival (OS) were more than 6 months up to now.

### 2.3 Case 3

**Figure 3:**
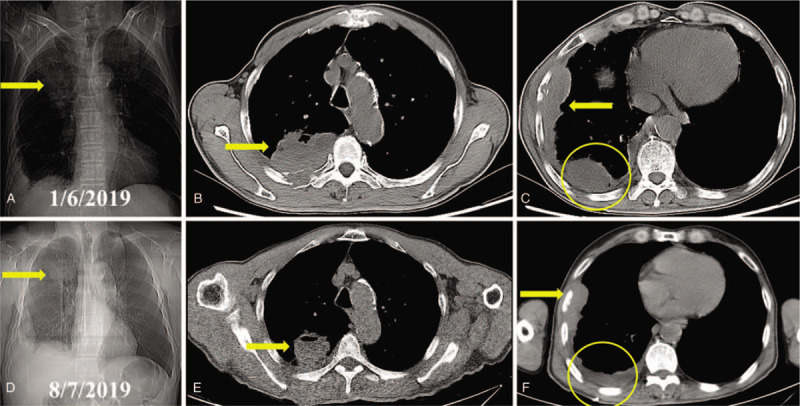
Radiological images of the thoracic lesions (indicated by arrow and circle) before and after systemic treatment in case 3. A. The X-ray showed a bulky mass located in right upper thorax. B. Computed tomography revealed 1 of the pulmonary tumors invading adjacent ribs. C. The other thoracic tumors with osteolytic rib destruction and thickened pleura were shown. D. X-ray indicated a smaller lesion in right upper thorax. E. The tumor showed partial response after 6 months of treatment. E. The other lesions and osteolytic ribs also revealed partial response. CT = computed tomography, PR = partial response.

A 69-year-old male was admitted because of chest stiffness and loss of body weight in the previous 2 months in January 2019. X-ray and CT showed thoracic lesions in right upper lobe invading ribs with pleural effusion, and one of the tumors was about 68 mm × 40 mm in size (Fig. 3). He had a smoking history of 60 pack-years. Then he was diagnosed with systemically disseminated PSC (T3NxM1, IV) using CT-guided percutaneous biopsy. First-line carboplatin, nab-paclitaxel, and apatinib (425 mg, daily), in addition to zoledronic acid were initiated timely. After 3 cycles of chemotherapy, the patient refused further intravenous treatment for financial reasons. Then apatinib (850 mg, daily) was continued as palliative therapy for another 1 month. However, the dosage was decreased to 425 mg daily thereafter because of Grade 3 leukocytopenia and hand-foot rash, although they were alleviated quickly after proper treatment. Encouragingly, both the osteolytic rib destruction and pulmonary mass of showed partial response nearly 7 months after the treatment.

## 3 Discussion

The incidence of PSC is nearly 0.5% of the NSCLC and 48% of them are staged IV at presentation, with a median OS of 5.8 months for stage III, and 5.4 months for stage IV patients.^[4]^ Because of its rarity and heterogeneity, the treatment and prognosis of PSC have not been clearly described, lacking reliable efficacy-related biomarkers.

Previous reports regarding the treatment of PSC has been listed in Table 1, which shows that the efficacy of surgery combined with chemotherapy/chemoradiotherapy for PSC patients is quite limited.^[5–15]^ PSC behaves in an aggressive way even in stage I-II compared to other subtypes of NSCLC.^[10]^ It represents a high risk for postoperative relapse,^[16]^ even in stage I after R0 resection.^[17]^ Because of its aggressive behavior, extended resection (bilobectomy, pneumonectomy, or even chest wall resection) are required, although the dismal prognosis still questions the role of surgery in PSC.

**Table 1:**
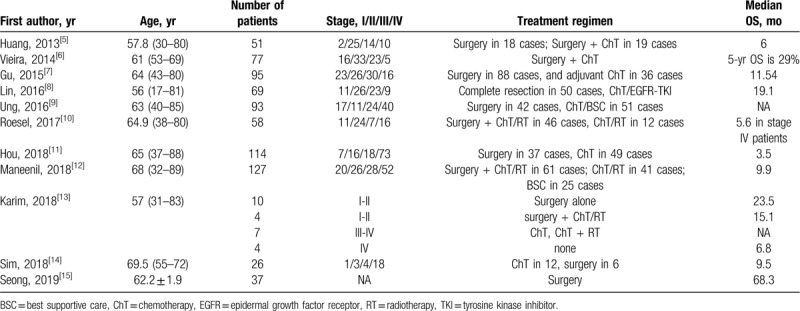
Previous reports about the therapeutic regimens for pulmonary sarcomatoid carcinoma.

On the other hand, platinum-based chemotherapy is reported to be associated with a significant 8% decrease of mortality in advanced PSC patients.^[18]^ Nevertheless, the overall response of locally advanced or metastatic cases to first-line chemotherapy is limited with a progression rate of 72%, meanwhile, the median time to progression and OS are 2.7 and 4.3 months, respectively.^[9]^ Furthermore, neither neoadjuvant nor adjuvant chemotherapy improves the survival of early-stage PSC patients.^[8]^

The high incidence of resistance to chemotherapy emphasizes the need of new strategies for the treatment of PSC. Due to high tumor mutation burden (TMB) and great prevalence for PD-L1 and 2 in PSC,^[18–21]^ immunotherapy (immune checkpoint inhibitors) shows encouragingly enduring efficacy in PSC patients (Table 2).^[22–25]^ Furthermore, the registered trials of immunotherapy in PSC is listed in Table 3.

**Table 2:**
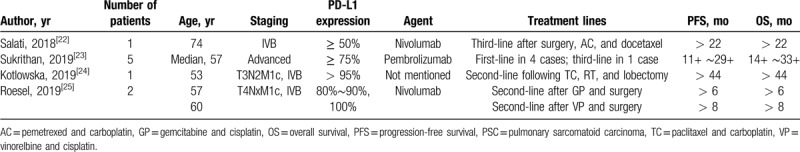
Case reports of immunotherapy for pulmonary sarcomatoid carcinoma.

**Table 3:**
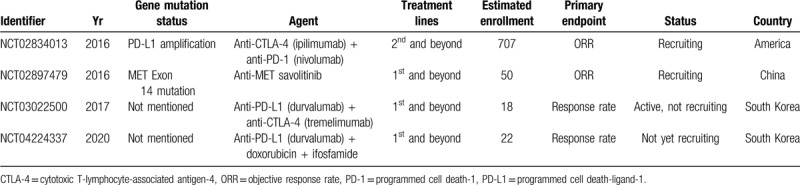
The registered trials of immunotherapy and targeted therapy for PSC patients.

One of the limitations of anti-angiogenic treatment is the inevitable drug-resistance, as shown in Case 1. Genetic alterations in PSC suitable for targeted therapy are poorly known by its rarity. Next-generation sequencing enables genome-wide molecular profiling of PSC regarding specific signal pathways of tumorigenesis, which is critical to pave the way to new treatment strategies. Anaplastic lymphoma receptor tyrosine kinase gene (ALK) and MNNG HOS transforming gene (MET) seem to act synergistically in PSC.^[26]^ In addition, KRAS and MET mutations may contribute to PSC tumorigenesis, and the epithelial mesenchymal transition pathway may play a key role in sarcomatoid transformation.^[27]^ Moreover, potentially targetable genomic alterations and intermediate or high TMB are identified in most PSC cases.^[28]^ The activation of epithelial mesenchymal transition drives PSC phylogeny in vivo, and dasatinib reverts the sarcomatoid-associated phenotype efficiently.^[29]^ In addition, TP53 and KRAS mutations are the most common genetic alterations in PSC, and KRAS mutation is a prognostic biomarker.^[30,31]^

## 4 Conclusions

First-line nab-paclitaxel/carboplatin plus oral apatinib showed limited short-term efficacy in advanced PSC. Besides the popular immune checkpoint inhibitors, more promising strategies for the treatment of PSC are still needed.

## Author contributions

**Conceptualization:** Feng-Wei Kong, Wei-Min Wang.

**Data curation:** Wei-Min Wang.

**Funding acquisition:** Wen-Bin Wu, Xiang Wang.

**Methodology:** Lei Liu, Long-Bo Gong.

**Resources:** Miao Zhang, Xiang Wang.

**Writing – original draft:** Feng-Wei Kong, Long-Bo Gong, Wen-Bin Wu.

**Writing – review & editing:** Lei Liu, Wen-Bin Wu, Miao Zhang.
